# Informal Caregiving, Loneliness and Social Isolation: A Systematic Review

**DOI:** 10.3390/ijerph182212101

**Published:** 2021-11-18

**Authors:** André Hajek, Benedikt Kretzler, Hans-Helmut König

**Affiliations:** Hamburg Center for Health Economics, Department of Health Economics and Health Services Research, University Medical Center, Hamburg-Eppendorf, 20246 Hamburg, Germany; b.kretzler.ext@uke.de (B.K.); h.koenig@uke.de (H.-H.K.)

**Keywords:** informal caregiving, loneliness, private caregiving, social exclusion, social isolation, spousal caregiving

## Abstract

*Background:* Several empirical studies have shown an association between informal caregiving for adults and loneliness or social isolation. Nevertheless, a systematic review is lacking synthesizing studies which have investigated these aforementioned associations. Therefore, our purpose was to give an overview of the existing evidence from observational studies. *Materials and Methods:* Three electronic databases (Medline, PsycINFO, CINAHL) were searched in June 2021. Observational studies investigating the association between informal caregiving for adults and loneliness or social isolation were included. In contrast, studies examining grandchild care or private care for chronically ill children were excluded. Data extractions covered study design, assessment of informal caregiving, loneliness and social isolation, the characteristics of the sample, the analytical approach and key findings. Study quality was assessed based on the NIH Quality Assessment Tool for Observational Cohort and Cross-Sectional Studies. Each step (study selection, data extraction and evaluation of study quality) was conducted by two reviewers. *Results:* In sum, twelve studies were included in our review (seven cross-sectional studies and five longitudinal studies)—all included studies were either from North America or Europe. The studies mainly showed an association between providing informal care and higher loneliness levels. The overall study quality was fair to good. *Conclusion*: Our systematic review mainly identified associations between providing informal care and higher loneliness levels. This is of great importance in assisting informal caregivers in avoiding loneliness, since it is associated with subsequent morbidity and mortality. Moreover, high loneliness levels of informal caregivers may have adverse consequences for informal care recipients.

## 1. Introduction

Remaining in familiar environments is often important for individuals in late life [1,2]. Therefore, home care is often preferred [3,4]. As the number of individuals needing care is likely to increase due to reasons of demographic ageing, home care is of great importance.

A key part of home care is the provision of informal care. This can be defined as the provision of private care for relatives, friends or neighbors in frequent need of care, including tasks such as personal care or simply assistance with the household [5]. A large body of evidence exists clearly demonstrating an association between informal caregiving and adverse health outcomes (such as decreased mental health, e.g., [6,7,8]).

Drawing on the caregiver stress model proposed by Pearlin et al. [9], informal caregiving can include several stressors such as burden [10]. These stressors can contribute to feelings of social isolation or loneliness [11]. Some studies have examined loneliness or social isolation in informal caregivers (e.g., [12,13,14,15]), partly demonstrating a link between provision of informal care and increased loneliness. This is plausible given the fact that informal caregiving can reduce the time available for family and friends due to reasons of prioritizing [16]—which can result in loneliness or isolation. Nevertheless, informal caregiving can also contribute to an increased size of social networks (e.g., by establishing contacts with other informal caregivers) and may therefore reduce feelings of loneliness or social isolation. Since a systematic review systematically synthesizing evidence regarding the association between informal caregiving (provided for adults) and loneliness or social isolation based on observational studies is lacking, our aim was to fill this gap in knowledge. Knowledge about this association may help to reduce these factors—which in turn, is of relevance since they are associated with several chronic illnesses, decreased perceived life expectancy [17,18] and reduced actual longevity [19,20].

It should be noted that loneliness and social isolation are related but distinct concepts [21]. For example, previous research showed a Pearson correlation of about five between loneliness and perceived social isolation [17]. While loneliness refers to the feeling that one’s social network is of a poorer quality or is smaller than desired [22,23], perceived social isolation refers to the feeling that one does not belong to society [22,23]. They also differ in their correlates and consequences (for further details, please see [17]). Both have in common the fact that they refer to social needs [24].

## 2. Methods

The methodology of this review satisfied the Preferred Reporting Items for Systematic Reviews and Meta-Analysis guidelines [25]. Additionally, this review is registered with the International Prospective Register of Systematic Reviews (PROSPERO, registration number: CRD42020193099). Moreover, a study protocol has been published [26].

### 2.1. Search Strategy and Selection Criteria

In June 2021, a systematic literature search was conducted in three databases (PubMed, PsycINFO, and CINAHL). The search query for PubMed is described in Table 1. A two-step process was used involving: 1. title/abstract screening and 2. full-text screening (independently by two reviewers (AH and BK). Additionally, a hand search was performed. Discussions were used when disagreements occurred. This approach was also used for data extraction and assessment of study quality.

Inclusion criteria were:

- cross-sectional and longitudinal observational studies analyzing the association between informal caregiving for adults (i.e., ≥18 years) and loneliness or social isolation
- operationalization of main variables with established tools
- studies in English or German language
- published in a peer-reviewed, scientific journal

In contrast, exclusion criteria were:

- studies examining grandchild care (e.g., [27,28])
- studies examining private care for chronically ill children
- studies exclusively using samples with a specific disorder among the caregivers (e.g., studies solely including caregivers with specific disorders)
- Prior to the final eligibility criteria, a pre-test was conducted (with a sample of 100 title/abstracts). Nevertheless, it should be emphasized that our criteria remained unchanged.

### 2.2. Data Extraction and Analysis

One reviewer (BK) carried out the data extraction, cross-checked by a second reviewer (AH). The data extraction covered the design of the study, operationalization of key variables (informal caregiving and loneliness/social isolation), characteristics of the sample, analytical approach, and important results. 

### 2.3. Assessment of Study Quality/Risk of Bias

The study quality was assessed using the NIH Quality Assessment Tool for Observational Cohort and Cross-Sectional Studies [29]. It is a well-known and widely used tool when dealing with observational studies (e.g., [30,31]). 

## 3. Results

### 3.1. Overview of Included Studies

**Figure 1:**
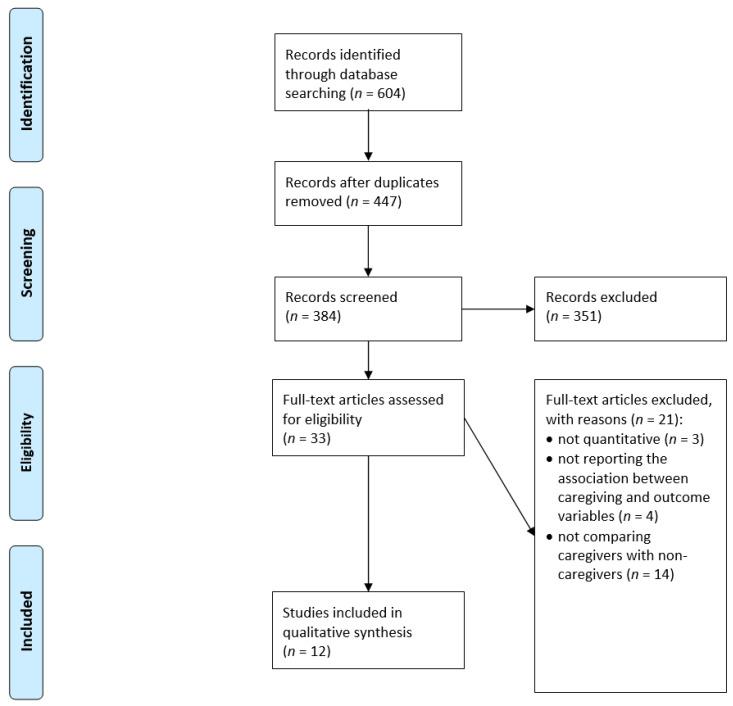
Flow Chart.

Figure 1 displays the selection process. In sum, *n* = 12 studies were included in our review [11,14,32,33,34,35,36,37,38,39,40,41]. The main findings are displayed in Table 2 (if given, adjusted results are shown in Table 2). Data came from North America (*n* = 5, all studies from the United States), and Europe (*n* = 7 studies, with three studies from Germany, one study from Norway, one study from Sweden, one study from the United Kingdom, and one study using data from Austria, Belgium, the Czech Republic, Denmark, Estonia, France, Germany, Italy, Luxembourg, Spain, and Switzerland). While seven studies were cross-sectional [32,33,34,35,37,40,41], five studies had a longitudinal design [11,14,36,38,39]. Among the longitudinal studies, the number of waves used ranged from two to four waves. The period of observation ranged from three to twelve years.

Two studies only used versions of the De Jong Gierveld scale to quantify loneliness and two studies only used different versions of the UCLA loneliness scale to quantify loneliness. Moreover, one study used the Bude and Lantermann scale to quantify perceived social isolation and the De Jong Gierveld scale to quantify loneliness. The other studies used different tools or single item measures to quantify feelings of loneliness. Half of the studies used a dichotomous variable to quantify the presence of informal caregiving. The other studies examined spousal caregiving or distinguished between, for example, current caregiving, former caregiving and non-caregiving.

Among the longitudinal studies, two studies used specific panel regression models to exploit the longitudinal data structure and to reduce the challenge of unobserved heterogeneity [42]. Based on these panel regression models, consistent estimates can be derived [42].

The sample size ranged from 101 to 29,458 observations (in sum, 91,857 observations). The studies mainly examined middle-aged and older individuals (average age ranged from 45.0 years to 83.7 years across the studies). The proportion of women in the samples mainly ranged from about 50% to 60%, whereas two studies had about 70% of women. Further details are shown in Table 2.

In the next sections, the results are displayed as follows: 1. Informal caregiving and loneliness (cross-sectional studies, thereafter longitudinal studies), and 2. Informal caregiving and social isolation (cross-sectional studies, thereafter longitudinal studies).

### 3.2. Informal Caregiving and Loneliness

In sum, *n* = 11 studies examined the association between informal caregiving and loneliness (six cross-sectional studies and five longitudinal studies).

With regard to cross-sectional studies, four studies found an association between caregiving and increased levels of loneliness [33,35,37,41], whereas one study found no association between these factors [32]. Moreover, one study found an association between caregiving and a decreased likelihood of loneliness [34]. However, this study was conducted during the COVID-19 pandemic.

With regard to longitudinal studies, three studies found an association between caregiving and increased loneliness levels [11,36,39], whereas two studies did not identify significant differences [14,38]. One of the three studies which found significant differences only found these among men, but not women [11].

### 3.3. Informal Caregiving and Social Isolation

In sum, *n* = 2 studies examined the association between informal caregiving and social isolation (one cross-sectional study and one longitudinal study). Both studies did not find an association between these factors [11,40]. It should be noted that one of these studies examined both the association between informal caregiving and loneliness as well as between informal caregiving and social isolation [11].

### 3.4. Quality Assessment

The assessment of the study quality of the studies included in our review is displayed in Table 3. While some important criteria were achieved by all studies (e.g., clear aim of the study or valid assessments of important variables), a few other criteria were only partly (e.g., adjustment for covariates) or hardly ever met (e.g., sufficient response rate or small loss to follow-up). Nevertheless, the overall study quality was quite high (seven studies were rated as ‘good’ and five studies were rated as ‘fair’; none of the studies were rated as ‘poor’).

## 4. Discussion

### 4.1. Main Findings

In summary, twelve studies were included in our review (seven cross-sectional studies and five longitudinal studies)—all included studies were either from North America or Europe. The studies mainly showed an association between providing informal care and higher loneliness levels. The overall study quality was fair to good. Such knowledge about an association between informal caregiving and loneliness is of great importance for targeting target individuals at risk of increased levels of loneliness, which in turn may assist in maintaining health.

### 4.2. Possible Mechanisms

Rather unsurprisingly, most of the studies included found an association between the provision of informal care and increased levels of loneliness. While only single studies (e.g., [43]) identified positive health consequences of informal caregiving, most of the studies showed harmful consequences of private care (e.g., on sleep [44], mental health or life satisfaction [7,8,44,45]). These harmful consequences may contribute to feelings of loneliness. More precisely, specific depressive symptoms such as anhedonia (inability to experience pleasure) may reduce motivation to perform social activities [46]. This in turn may result in feelings of loneliness. Furthermore, the reduced sleep quality caused by performing informal care may also inhibit physical and cognitive activities [44] which can ultimately contribute to reduced loneliness scores. Similarly, a reduced satisfaction with life can directly contribute to social withdrawal or feeling lonely [47].

Furthermore, the association between informal caregiving and increased loneliness may be explained by the fact that informal caregiving limits social contacts [48,49,50]. In turn, this may enhance emotions of loneliness caused by the restricted leisure time for social activities [51], caregiving burden or emotions such as guilt or resentment [48,49,50].

### 4.3. Comparability of Studies

Several factors limit the comparability of the studies included. For example, both loneliness and social isolation were quantified using different tools. None of the studies examined the association between informal caregiving and objective social isolation. Informal caregiving was also assessed differently between the studies. More than half of the studies included used cross-sectional data. Out of the five longitudinal studies, only two used specific panel regression models. Such models are required to produce consistent estimates [42]. With regard to cultural differences, the included studies exclusively referred to data from North America or Europe.

### 4.4. Gaps in Knowledge and Guidance for Future Research

Our current systematic review determined various gaps in our current knowledge. First, more longitudinal studies are needed to identify the impact of caregiving on loneliness and social isolation. Second, more studies using data from nationally representative samples are desirable. Third, caregiving types could be taken into consideration in future studies (e.g., from pure supervision to performing nursing care services [43,52]). Fourth, the relationship between caregiver and care-recipient (e.g., spousal caregiving vs. parental caregiving or inside household caregiving vs. outside household caregiving) should be taken into consideration. Fifth, the care-recipients should be clearly characterized (e.g., care recipient with cancer vs. care recipient with dementia)—if data are available. Sixth, future research should ideally use established instruments such as the De Jong Gierveld scale or the UCLA loneliness scale. Seventh, many more studies should also consider the impact of caregiving on (perceived and objective) social isolation. Eighth, research from other areas of the world (other than Europe and North America) is urgently needed. Ninth, the underlying mechanisms in the association between caregiving and loneliness as well as social isolation should be explored. Tenth, the association between caregiving and loneliness/social isolation should be further explored during (or after) the COVID-19 pandemic. Eleventh, subgroup analyses (e.g., stratified by gender) are desirable.

### 4.5. Strengths and Limitations

This is the first systematic review regarding the association between informal caregiving and loneliness/social isolation. The important steps were conducted by two reviewers. A meta-analysis was not performed due to study heterogeneity. Since we restricted our search to articles published in peer-reviewed articles, some important studies may be excluded from this review. However, it should be noted that a certain quality of the studies is ensured by this inclusion criterion.

## 5. Conclusions

In conclusion, our systematic review mainly identified associations between providing informal care and higher loneliness levels. This is of great importance in assisting informal caregivers in avoiding loneliness, since it is associated with subsequent morbidity and mortality. Moreover, high loneliness levels of informal caregivers may have adverse consequences for informal care recipients (e.g., in terms of earlier admission to nursing homes or decreased informal care quality). Thus, avoiding higher loneliness levels of individuals providing informal care may, more generally, assist in improving the relationship between informal caregivers and informal care recipients—which could be examined in future studies. This may also contribute to successful ageing in both informal caregivers and care recipients.

## Figures and Tables

**Table 1:** Search strategy (Medline search algorithm).

#	Search Term
#1	Informal Careg *
#2	Family careg *
#3	Private careg *
#4	Spousal careg *
#5	Parental careg *
#6	#1 OR #2 OR #3 OR #4 OR #5
#7	Lonel *
#8	Social isolation
#9	Social exclusion
#10	#7 OR #8 OR #9
#11	#6 AND #10

Table notes: The asterisk (*) is a truncation symbol. The number sign (#) refers to the search order.

**Table 2:** Study overview and important findings.

First Author	Country	Assessment of Informal Care	Assessment of Loneliness or Social Isolation	Study Type	Sample Characteristics	Sample Size; Age; Females in Total Sample	Results
Beach (2021) [32]	United States	dichotomous (yes/no)	increase in loneliness due to COVID-19 (yes/no)	cross-sectional	family caregivers and non-caregivers	*n* = 3509; M: 58.5, SD: 16.2; 18–100; 69.5%	Regarding a *t*-test, there were no differences in the changes of loneliness due to COVID-19 between caregivers and non-caregivers.
Beeson (2003 [33])	United States	dichotomous (yes/no)	UCLA Loneliness Scale (20 items)	cross-sectional	Alzheimer’s disease caregiving spouses and non-caregiving spouses	*n* = 101; M: 75.8, SD: 8.4; 58.4%	According to a *t-*test, caregiving spouses had significantly higher loneliness levels than non-caregiving spouses (37.4 vs. 33.1, *p* < 0.05).
Brandt (2021) [34]	Germany	providing assistance which is necessary for others (yes/no)	missing company (yes/no)	cross-sectional	community-dwelling individuals aged 40 years and older	*n* = 353; M: 58.9, 40–91; 72.0%	According to logistic regression, people who provided assistance were significantly less likely to miss company (ß = −0.17, *p* < 0.05).
Ekwall (2005) [35]	Sweden	dichotomous (yes/no)	loneliness (three items rated on four-point-scale)	cross-sectional	population-based sample consisting of individuals aged 75 years and older	*n* = 4278; M: 83.7, SD: 5.5; 60.6%	Feelings of loneliness were more frequent among non-caregivers (e.g., recurrent feelings of loneliness: 10.9% vs. 5.8%, *p* < 0.001).
Gallagher (2020) [36]	United Kingdom	dichotomous (yes/no)	loneliness during the last three weeks rated on a three-point scale	longitudinal (two waves from 2017 to 2020)	Understanding Society/UK Household Longitudinal Study	*n* = 7537; M: 48.4, SD: 17.2; 53.1%	Regarding F-tests, carers had significantly higher levels of loneliness before COVID-19 (8.0% vs 7.5%, *p* < 0.001) and during COVID-19 (8.2% vs 7.1%, *p* < 0.05).
Hajek (2019) [14]	Germany	dichotomous (yes/no)	De Jong Gierveld Loneliness Scale (eleven items)	longitudinal (four waves from 2002 to 2014)	German Ageing Survey	*n* = 21,762; M: 62.3, SD: 11.4, 40–95; 49.6%	According to fixed-effects regression, there were no significant differences in loneliness.
Hansen (2015) [37]	Norway	non-caregiver; in-household caregiver; out-of-household caregiver	De Jong Gierveld Loneliness Scale (eight items)	cross-sectional	Norwegian Life Course, Ageing and Generation study	*n* = 11,047; M: 45.0, SD: 11.0, 25–64; 51.2%	Regression analysis showed that in-household caregivers (compared to non-caregivers) have increased levels of loneliness (ß = 0.13, *p* < 0.05). In addition, the interactions in-household caregiver x part-time employment (ß = 0.27, *p* < 0.05) and in-household caregiver x non-working (ß = 0.20, *p* < 0.05) were also related to increased loneliness.
Hawkley (2020) [38]	United States	spousal caregiver (yes/no)	UCLA Loneliness Scale (three items)	longitudinal (two waves from 2010 to 2015)	National Social Life, Health and Aging Project	*n* = 970; ≤64: 32.0% 65–74: 46.8% 75–84: 19.9% ≥85: 1.5%; 50.0%	*t*-tests revealed no significant differences between caregivers and non-caregivers.
Robinson-Whelen (2001) [39]	United States	current caregiver; former caregiver; non-caregiver	New York University Loneliness Scale (three items)	longitudinal (four waves during four years)	caregivers and control participants	*n* = 143; M: 69.3, SD: 8.9 Female: not specified	Regarding the graphical presentation, both former and current caregivers had higher levels of loneliness than a control group.
Robison (2009) (Robison et al., 2009) [40]	United States	dichotomous (yes/no)	going out too little	cross-sectional	Connecticut Long-Term Care Needs Assessment	*n* = 4041; M: 71.5; 61.1%	Logistic regression did not reveal a significant association between caregiving and social isolation.
Wagner (2018) [41]	Austria, Belgium, the Czech Republic, Denmark, Estonia, France, Germany, Italy, Luxembourg, Spain, and Switzerland	spousal caregiver (yes/no)	UCLA Loneliness Scale (three items)	cross-sectional	Survey of Health, Ageing and Retirement in Europe	*n* = 29,458; M: 64.5 SD: 9.4 30–95; 50.4%	According to regression analysis, spousal care was correlated with increased levels of loneliness (ß = 0.12, *p* < 0.001).
Zwar (2020) [11]	Germany	not reporting care at baseline but having started to do so at follow-up	loneliness: De Jong Gierveld Loneliness Scale (six items)social isolation: instrument from Bude and Lantermann (2006) (Bude and Lantermann, 2006) (four items)	longitudinal (two waves from 2014 to 2017)	German Ageing Survey	*n* = 8658; M: 65.9 SD: 10.6; 54.5%	Fixed-effects regression found caregiving to be significantly associated with higher levels of loneliness among men (ß = 0.93, *p* < 0.01), but not with social isolation.

**Table 3:** Quality Assessment.

Paper Author and Date	1. Was the Research Question or Objective in This Paper Clearly Stated?	2. Was the Study Population Clearly Specified and Defined?	3. Was the Participation Rate of Eligible Persons at Least 50%?	4. Were all the Subjects Selected or Recruited from the Same or Similar Populations (Including the Same Time Period)? Were Inclusion and Exclusion Criteria for Being in the Study Prespecified and Applied Uniformly to All Participants?	5. Was a Sample Size Justification, Power Description, or Variance and Effect Estimates Provided?	6. For the Analyses in This Paper, Were the Exposure(s) of Interest Measured Prior to the Outcome(s) Being Measured? (if not Prospective Should Be Answered as ‘no’, Even Is Exposure Predated Outcome)	7. Was the Timeframe Sufficient so That One Could Reasonably Expect to See an Association between Exposure and Outcome if It Existed?
Beach (2021)) [32]	Yes	Yes	No (40%)	Yes	No	No (cross-sectional)	No (cross-sectional)
Beeson (2003 [33])	Yes	Yes	Not reported	Yes	No	No (cross-sectional)	No (cross-sectional)
Brandt (2021) [34]	Yes	Yes	Not reported	Yes	No	No (cross-sectional)	No (cross-sectional)
Ekwall (2005) [35]	Yes	Yes	Yes (52.8%)	Yes	Yes	No (cross-sectional)	No (cross-sectional)
Gallagher (2020) [36]	Yes	Yes	Not reported	Yes	No	No (simultaneously)	Yes
Hajek (2019) [14]	Yes	Yes	No (e.g., 38% response rate in wave 2)	Yes	No	No (simultaneously)	Yes
Hansen (2015) [37]	Yes	Yes	No (43.2%)	Yes	No	No (cross-sectional)	No (cross-sectional)
Hawkley (2020) [38]	Yes	Yes	Yes (e.g., 87% in wave 2)	Yes	No	No (simultaneously)	Yes
Robinson-Whelen (2001) [39]	Yes	Yes	Not reported	Yes	No	No (simultaneously)	Yes
Robison (2009) (Robison et al., 2009) [40]	Yes	Yes	No (29%)	Yes	No	No (cross-sectional)	No (cross-sectional)
Wagner (2018) [41]	Yes	Yes	Not reported	Yes	No	No (cross-sectional)	No (cross-sectional)
Zwar (2020) [11]	Yes	Yes	No (e.g., 27.1% in wave 5)	Yes	No	No (simultaneously)	Yes
**Paper Author and Date**	**8. For exposures that can vary in amount or level, did the study examine different levels of the exposure as related to the outcome (e.g., categories of exposure, or exposure measured as continuous variable)?**	**9. Were the exposure measures (independent variables) clearly defined, valid, reliable, and implemented consistently across all study participants?**	**10. Was the exposure(s) assessed more than once over time?**	**11. Were the outcome measures (dependent variables) clearly defined, valid, reliable, and implemented consistently across all study participants?**	**12. Was loss to follow-up after baseline 20% or less?**	**13. Were key potential confounding variables measured and adjusted statistically for their impact on the relationship between exposure(s) and outcome(s)?**	**Overall quality judgement**
Beach (2021)) [32]	Dichotomous	Yes	Not applicable	Yes	Not applicable	No	Good
Beeson (2003 [33])	Dichotomous	Yes	Not applicable	Yes	Not applicable	No	Fair
Brandt (2021) [34]	Dichotomous	Yes	Not applicable	Yes	Not applicable	Yes	Fair
Ekwall (2005) [35]	Dichotomous	Yes	Not applicable	Yes	Not applicable	No	Fair
Gallagher (2020) [36]	Dichotomous	Yes	Yes	Yes	Not reported	No	Fair
Hajek (2019) [14]	Dichotomous	Yes	Yes	Yes	Not reported	Yes	Good
Hansen (2015) [37]	Three categories	Yes	Not applicable	Yes	Not applicable	Yes	Good
Hawkley (2020) [38]	Dichotomous	Yes	Yes	Yes	Not reported	Yes	Good
Robinson-Whelen (2001) [39]	Three categories	Yes	Yes	Yes	Not reported	No	Fair
Robison (2009) (Robison et al., 2009) [40]	Dichotomous	Yes	Not applicable	Yes	Not applicable	Yes	Good
Wagner (2018) [41]	Dichotomous	Yes	Not applicable	Yes	Not applicable	Yes	Good
Zwar (2020) [11]	Dichotomous	Yes	Yes	Yes	No (e.g., follow-up rate from the panel sample was 63% in wave 6)	Yes	Good

## Data Availability

Not applicable.
